# Impact of interventional periodontal therapy on the longevity of failing dental implants: A retrospective analysis

**DOI:** 10.4317/medoral.28043

**Published:** 2026-01-24

**Authors:** Georgios S Chatzopoulos, Larry F Wolff

**Affiliations:** 1 Department of Developmental and Surgical Sciences, Division of Periodontology, School of Dentistry, University of Minnesota, 515 Delaware Street SE, Minneapolis, MN,55455, USA; 2 Department of Preventive Dentistry, Periodontology and Implant Biology, School of Dentistry, Aristotle University of Thessaloniki, 54124, Thessaloniki, Greece

## Abstract

**Background:**

Biological complications such as peri-implantitis present a significant challenge to the long-term stability of dental implants, often leading to failure if left untreated. While a history of periodontitis is a known risk factor, there is a lack of real-world data quantifying the "survival extension" provided by therapeutic interventions. This study aimed to evaluate the efficacy of surgical and non-surgical "rescue" therapies in extending the lifespan of dental implants that ultimately failed.

**Material and Methods:**

A retrospective cohort analysis was conducted using the multi-institutional BigMouth dental data repository, examining patients treated between 2011 and 2022. The study identified 725 failed implants, which were stratified into two clusters: A "Rescue Therapy" group (n=130) that received active intervention (e.g., scaling, flap surgery, bone grafting) and a "No Rescue" control group (n=595) that received no intervention prior to failure. The primary outcome was time-to-failure, calculated in months. A subgroup analysis of implants surviving beyond 12 months was performed to isolate late-stage biological failures.

**Results:**

The Rescue Therapy group demonstrated a statistically significant survival advantage over the No Rescue group. In the subgroup of implants surviving the initial osseointegration phase (&gt;12 months), those receiving rescue therapy achieved a median survival of 91.1 months, compared to 40.9 months for untreated implants (p&lt;0.001). This differential equates to a median "survival extension" of 50.2 months (approximately 4.2 years) attributable to active intervention. Non-smokers showed a trend toward higher utilization of rescue therapies.

**Conclusions:**

Interventional periodontal therapy significantly extends the functional lifespan of failing dental implants compared to non-intervention. For late-stage failures, rescue therapy provides a clinically meaningful survival extension of over four years. These findings advocate for an aggressive interventional approach to manage peri-implant biological complications, delaying explantation and maintaining function.

## Introduction

Dental implants have become a predictable and widely accepted treatment modality for the rehabilitation of fully and partially edentulous patients, demonstrating high long-term survival rates exceeding 95% over 10 years (1 , 2). However, despite this initial success, biological complications such as peri-implant mucositis and peri-implantitis remain prevalent challenges that threaten long-term stability (3). Peri-implantitis, characterized by progressive loss of supporting bone, is a pathological condition that, if left untreated, often leads to implant failure and explantation (4). As the number of implants placed globally continues to rise, the burden of managing these biological failures is increasing, necessitating effective therapeutic strategies to arrest disease progression and extend implant function (5).

A history of periodontitis is a well-established risk factor for both implant failure and the development of peri-implantitis (6). Patients with a history of moderate to severe periodontitis exhibit significantly higher risks of marginal bone loss and late implant failure compared to periodontally healthy individuals (7 , 8). Studies indicate that susceptibility to periodontal disease may translate to a "peri-implantitis prone" phenotype, where the host response to bacterial challenge is compromised (9). Consequently, these high-risk populations require more rigorous surveillance and intervention protocols to maintain peri-implant health (10).

Supportive periodontal therapy (SPT) and regular maintenance are critical for preventing biological complications. Consistent evidence demonstrates that patients who adhere to individualized maintenance programs-typically every 3 to 6 months-show significantly lower rates of peri-implant disease and implant loss compared to non-compliant individuals (11 , 12). Maintenance visits allow for the early detection of mucositis and the removal of biofilm, which is the primary etiological factor in peri-implant disease (13). Conversely, irregular maintenance or "compliance drift" is strongly associated with increased probing depths, bleeding on probing, and eventual bone loss (14).

When prevention fails and peri-implantitis develops, the clinical management becomes complex and unpredictable. Current treatment modalities generally fall into two categories: Non-surgical therapy (including mechanical debridement, scaling and root planing, and the use of antiseptics or antibiotics) and surgical therapy (including open flap debridement, resective surgery, and regenerative procedures using bone grafts) (15). While non-surgical therapy is often the first line of defense for mucositis, it has shown limited efficacy in arresting deep peri-implant lesions or re-establishing osseointegration in cases of advanced bone loss (16).

Surgical interventions are often advocated for advanced peri-implantitis to modify the implant surface and regenerate lost support. Studies suggest that surgical therapy, particularly when combined with strict supportive care, can achieve disease resolution in approximately 60% of cases and arrest bone loss in up to 70% of affected sites (17). However, the long-term prognosis of "rescued" implants remains uncertain. Residual deep pockets and advanced bone loss following therapy are strong predictors of disease recurrence, leading some clinicians to question whether these interventions provide a meaningful extension of implant life or merely delay inevitable explantation (18 , 19).

There is a significant gap in the literature regarding the "survival extension" provided by these rescue therapies in a real-world setting. Most existing studies are prospective clinical trials with small sample sizes and strict inclusion criteria that may not reflect the general population (20). Furthermore, few large-scale retrospective studies have quantified exactly how long a failing implant can be retained following active intervention compared to one that receives no treatment. Understanding this "functional extension" is crucial for clinicians when advising patients on the cost-benefit ratio of treating versus replacing a failing implant (21).

To address this knowledge gap, this study utilizes the BigMouth dental data repository to analyze a massive cohort of failed implants. Unlike previous studies that focus on "success" rates, this investigation specifically targets the "failing" cohort to determine if intervention alters the trajectory of failure. By leveraging a large-scale dataset, we can control for numerous patient-level variables, including systemic conditions like diabetes and smoking, which are known to compound the risk of failure (22 , 23).

The aim of this retrospective cohort study was to evaluate the efficacy of rescue therapy (non-surgical and surgical) in extending the lifespan of dental implants that ultimately failed. The specific objectives were to compare the time-to-failure between implants that received active periodontal intervention versus those that did not, and to identify patient characteristics associated with the decision to pursue rescue therapy. The null hypothesis tested was that there is no difference in the survival time of failing implants regardless of whether rescue therapy was performed. This study is novel in its use of "survival extension" as a metric, providing real-world data on the tangible value of treating peri-implant disease in compromised sites.

## Material and Methods

Study Design and Data Source

This study was designed as a retrospective cohort analysis and follows the Strengthening the Reporting of Observational Studies in Epidemiology (STROBE) guidelines (24). The dataset was obtained from the BigMouth dental data repository (Harvard University; University of Texas Health; The University of California, San Francisco; University of Colorado; Loma Linda University; University of Buffalo; The University of Iowa; The University of Minnesota; and Tufts University), a multi-institutional electronic health record (EHR) network. This repository aggregates partially identified dental and medical data from several university-based dental clinics across the United States.

The study protocol was reviewed by the University of Minnesota Institutional Review Board (IRB) and an approval waiver was granted (#STUDY00016865, 10/10/2022). In addition, the BigMouth Consortium for Oral Health Research and Informatics approved the investigation ensuring compliance with the Helsinki Declaration's ethical guidelines. A waiver of informed consent was granted due to the retrospective nature of the study and the use of de-identified data.

Study Population

The initial cohort consisted of patients who underwent dental implant therapy at the participating university clinics between January 1, 2011, and December 31, 2022.

To address the specific research question regarding the efficacy of therapeutic intervention on failing implants, the following inclusion criteria were applied:

1. Implant Placement: Patients must have a recorded Current Dental Terminology (CDT) code for dental implant placement (D6010) within the study period.

2. Implant Failure: The study specifically isolated a cohort of "failed" implants, defined as those with a subsequent record of implant removal (CDT code D6100, or specific "Explantation" descriptions) linked to the same patient and tooth number.

3. Complete Records: Patients were required to have complete demographic data (age, gender) and medical history at the time of baseline.

Exclusion Criteria

- Implants with incomplete placement or removal dates.

- Patients &lt;18 years of age at the time of placement.

- Implants removed on the same day as placement (immediate surgical complications) were excluded from the longitudinal survival analysis.

Data Extraction and Clustering (Exposure Definition)

Data were extracted using structured query language (SQL) scripts to retrieve patient demographics, medical history, dental procedures, and medication records.

The primary independent variable was the receipt of "Rescue Therapy." The cohort of failed implants was stratified into two distinct clinical clusters based on the presence or absence of active periodontal/peri-implant therapeutic interventions during the maintenance phase (the interval between implant placement and implant removal):

- Cluster 1: Rescue Therapy Group. Defined as implants that received at least one active therapeutic intervention after placement and prior to removal. "Rescue" interventions were identified using specific CDT codes corresponding to:

Non-Surgical Therapy: Scaling and root planing (D4341, D4342) or localized scaling (D4346) in the implant quadrant.

Surgical Therapy: Gingival flap procedures (D4240, D4241), osseous surgery (D4260, D4261), bone replacement grafts (D4263, D4264), guided tissue regeneration (D4266, D4267), or gingivectomy (D4210, D4211).

Routine prophylactic procedures (D1110) and periodontal maintenance (D4910) were considered standard of care and did not qualify an implant for the "Rescue" cluster.

- Cluster 2: No Rescue Group (Control). Defined as implants that progressed from placement to failure without any recorded active therapeutic periodontal intervention (surgical or non-surgical) during the maintenance interval.

Study Variables and Outcome Measures

- Outcome Variable: The primary outcome was "Time to Failure" (Survival Time), calculated as the interval in months between the date of implant placement and the date of implant removal.

- Covariates: Patient-level variables included baseline age, gender, and self-reported race/ethnicity. Systemic health status was assessed using medical diagnosis codes (ICD-9/ICD-10) and medical history forms to identify conditions such as diabetes mellitus (Type 1 and 2), hypertension, and tobacco use (current smoker vs. non-smoker).

Statistical Analysis

Descriptive statistics were used to summarize the cohort characteristics. Continuous variables (e.g., age, time to failure) were expressed as means±standard deviations (SD) and medians with interquartile ranges (IQR). Categorical variables (e.g., gender, smoking status) were presented as frequencies and percentages.

To assess the efficacy of rescue therapy, the distribution of "Time to Failure" was compared between the Rescue Therapy and No Rescue clusters.

1. Normality Testing: The Shapiro-Wilk test was used to assess the normality of the survival time distribution. As the data were non-normally distributed, non-parametric tests were employed.

2. Primary Analysis: The Mann-Whitney U test was utilized to compare the median survival time between the two clusters.

3. Subgroup Analysis (Late Failures): To control for "early failures" (failure to osseointegrate) where rescue therapy is rarely indicated or feasible, a secondary analysis was performed including only implants that survived &gt;12 months. This isolated the effect of intervention on biological complications (e.g., peri-implantitis).

A p-value of &lt;0.05 was considered statistically significant.

## Results

Patient and Implant Characteristics

A total of 725 failed dental implants were included in the analysis. The cohort was stratified into two groups: The Rescue Therapy Group (n=130), which received active periodontal intervention (e.g., Scaling and Root Planing, Flap Surgery, Bone Grafting) post-placement, and the No Rescue Group (n=595), which proceeded to failure without recorded therapeutic intervention.

Baseline demographic and systemic health characteristics were comparable between the two groups (Table 1).

Table 1

There were no statistically significant differences in age (p=0.63), gender (p=0.40), or systemic comorbidities such as diabetes (p=0.62) and hypertension (p=0.54). The prevalence of smoking was lower in the Rescue Therapy group (4.6%) compared to the No Rescue group (10.6%), though this approached but did not reach statistical significance (p=0.053), suggesting a trend where non-smokers may be more likely to receive or pursue rescue interventions.

Overall Time-to-Failure Analysis

Initial analysis compared the total lifespan of implants across both groups, inclusive of early failures (failure to osseointegrate). The distribution of time-to-failure was non-normal; therefore, non-parametric testing was utilized.

The Rescue Therapy group demonstrated a significantly longer median survival time compared to the No Rescue group. The median time to failure for the Rescue Therapy group was 88.8 months (IQR: 47.7-118.2), compared to 8.7 months (IQR: 3.5-31.4) for the No Rescue group. This difference of approximately 80.1 months was statistically significant (Mann-Whitney U=65,472.5, p&lt;0.001) (Table 2).

Table 2

Subgroup Analysis: Late Failures (&gt;12 Months)

To control for the confounding variable of early surgical failure (failure to osseointegrate), which typically occurs within the first year and precludes the opportunity for rescue therapy, a subgroup analysis was performed on implants surviving longer than 12 months. This isolated the efficacy of intervention on biological complications (e.g., peri-implantitis).

In this sub-cohort (n=374), the survival advantage of rescue therapy remained statistically significant. The Rescue Therapy group (n=121) exhibited a median survival of 91.1 months compared to 40.9 months in the No Rescue group (n=253).

**Figure 1:**
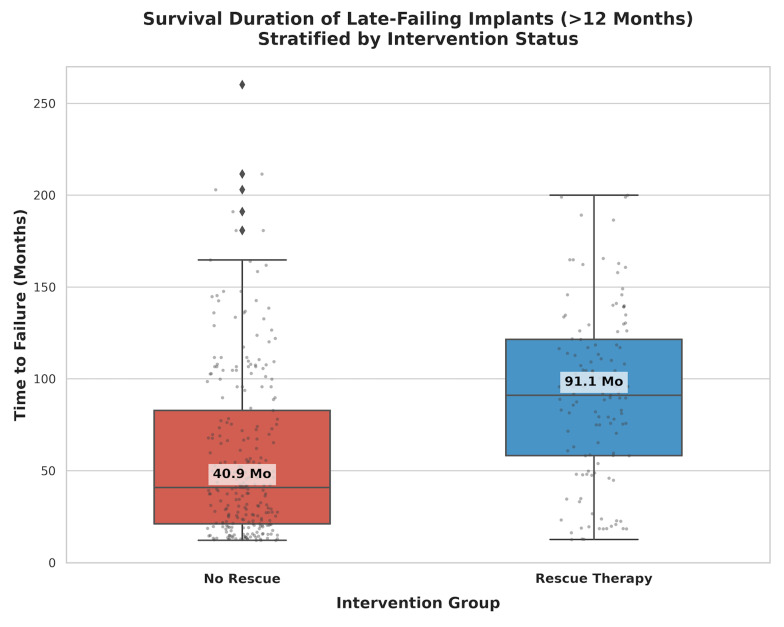
Boxplot of implant survival duration by intervention group (late failures). Distribution of time-to-failure (in months) for implants surviving &gt;12 months. The orange box represents the "Rescue Therapy" group, showing a significantly elevated median and interquartile range compared to the blue "No Rescue" group. Whiskers indicate the range excluding outliers. The median survival shifts from approx. 41 months to 91 months with intervention.

The calculated "Survival Extension" attributable to intervention in late-stage failures was 50.2 months (approx. 4.2 years). The distributions differed significantly (Mann-Whitney U=21,664.5, p&lt;0.001) (Table 3, Figure 1).

Table 3 1

## Discussion

**Figure 2:**
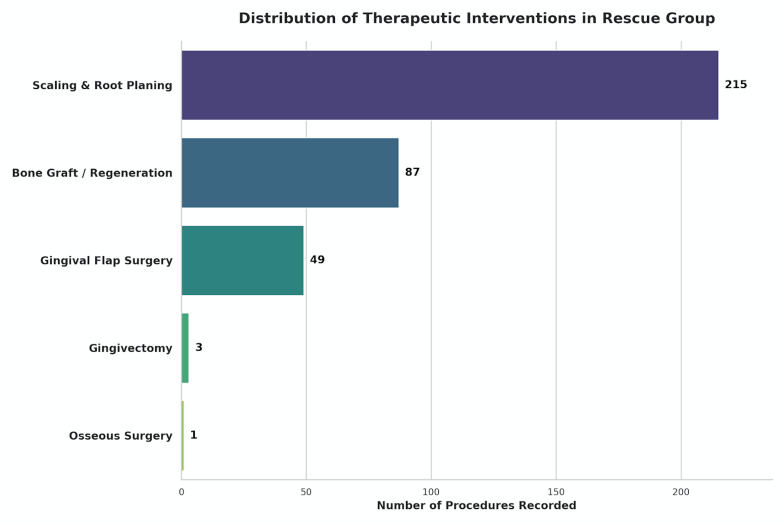
Frequency distribution of interventions.Frequency of specific rescue interventions utilized in the rescue therapy cohort. The most common intervention was scaling and root planing (D4341/D4342), followed by gingival flap surgery (D4240/D4241) and bone replacement grafts (D4263/D4265).

The primary aim of this large-scale retrospective cohort study was to evaluate the efficacy of "rescue" periodontal therapy -comprising both surgical and non-surgical interventions (Figure 2)- in extending the lifespan of failing dental implants. Utilizing the multi-institutional BigMouth dental data repository, this investigation specifically targeted a cohort of implants that ultimately failed, allowing for a direct comparison of survival trajectories between those that received intervention and those that did not. The results lead to the rejection of the null hypothesis; the data demonstrate that active therapeutic intervention significantly prolongs the functional life of failing implants. Specifically, in implants surviving the initial osseointegration phase (&gt;12 months), rescue therapy provided a median "survival extension" of approximately 50.2 months (4.2 years) compared to untreated failing implants.

2

Interpretation in the Context of Available Evidence

The findings of this study provide critical real-world data that complements existing prospective literature. While dental implants generally demonstrate high survival rates, biological complications remain a prevalent challenge, particularly in patients with a history of periodontitis (2 , 4). Previous systematic reviews have established that a history of periodontitis is a significant risk factor for both implant failure and peri-implantitis (5 , 8). The current study reinforces the importance of ongoing vigilance in these high-risk populations. By quantifying the "time-to-failure," our results underscore that even when an implant is destined to fail, the trajectory of that failure is modifiable.

The observed survival advantage in the "Rescue Therapy" group aligns with the consensus that supportive periodontal therapy (SPT) and maintenance are essential for implant longevity (3 , 13). Studies have consistently shown that adherence to maintenance programs reduces the incidence of peri-implant diseases (7 , 11). For instance, reduced compliance with maintenance has been associated with poorer peri-implant conditions and increased bone loss (12 , 14). Our data extends this narrative by suggesting that even after disease onset (i.e., when "maintenance" shifts to "rescue"), active intervention continues to offer tangible value.

The distinction between "early" and "late" failures in our analysis is crucial. Early failures are often attributed to surgical trauma, lack of primary stability, or infection preventing osseointegration, whereas late failures are predominantly biological (peri-implantitis) or biomechanical (1). By conducting a subgroup analysis of implants surviving beyond 12 months, we isolated the cohort where biological rescue is most biologically plausible. In this group, the extension of life was most pronounced. This finding supports recent perspectives on the surgical management of peri-implantitis, which advocate for intervention to arrest bone loss and modify the implant surface (15). While re-osseointegration remains unpredictable, the stabilization of the peri-implant environment clearly delays explantation.

Strengths and Limitations

A major strength of this study is the use of the BigMouth repository, which provided a massive, multi-center dataset representing "real-world" clinical practice rather than the controlled environment of a clinical trial. This allows for high external validity and the ability to control for numerous covariates (21). Furthermore, the use of "survival extension" as a primary outcome addresses a gap in the literature, which typically focuses on binary "success/failure" metrics rather than the duration of function in compromised sites.

However, several limitations inherent to retrospective designs must be acknowledged (24). First, the allocation to the "Rescue Therapy" group was not randomized, introducing potential selection bias. It is possible that patients who received rescue therapy were more compliant, had better financial means, or were treated by clinicians who perceived the implant as "salvageable," whereas implants in the "No Rescue" group may have been deemed hopeless earlier. Additionally, while we controlled for systemic factors like diabetes and smoking (22 , 23), the database relies on CDT and ICD codes, which may lack the granularity of clinical parameters such as probing depths or radiographic bone levels (17). Finally, the dataset does not distinguish between specific surgical techniques (e.g., resective vs. regenerative) with enough frequency to analyze the superiority of one specific modality over another (20).

Clinical Implications

The clinical relevance of these findings is significant for the day-to-day decision-making process. When a clinician encounters a failing implant, the decision to treat or explant is complex (18). Patients often inquire, "If we treat this, how much longer will it last?" Our data suggests that for late-stage failures, intervention can buy the patient an average of four additional years of function. This "functional extension" allows time for financial planning, delaying complex restorative transitions, or simply maintaining quality of life (10). Conversely, the rapid demise of untreated implants (median ~41 months in late failures) highlights the risks of a "watch and wait" approach (9 , 16).

## Conclusions

Within the limitations of this retrospective analysis, the following conclusions can be drawn:

- Interventional periodontal therapy ("rescue therapy") significantly extends the survival time of failing dental implants compared to those that receive no intervention.

- In implants that have successfully osseointegrated (survived &gt;12 months), active intervention is associated with a median survival extension of approximately 4.2 years.

- Non-smokers showed a trend toward higher utilization of rescue therapies, suggesting that patient-related factors influence the decision to treat.

These findings advocate for an aggressive interventional approach when managing peri-implant biological complications. While "rescue" therapy may not always result in permanent "cure" or "success" by strict definitions, it provides a clinically meaningful extension of implant function. Future prospective studies should aim to stratify this survival extension by specific surgical modalities (regenerative vs. resective) to further optimize treatment protocols for failing implants.

## Figures and Tables

**Table 1:** Table Baseline characteristics of included implants by intervention group.

Characteristic	No rescue group (n=595)	Rescue therapy group (n=130)	P-value
Age at Baseline (Years)			
Mean (SD)	57.3 (±12.9)	57.8 (±9.8)	0.63
Gender, n (%)			
Male	297 (49.9%)	59 (45.4%)	0.40
Female	298 (50.1%)	71 (54.6%)	
Systemic Health, n (%)			
Smoker (Current)	63 (10.6%)	6 (4.6%)	0.053
Diabetes	42 (7.1%)	7 (5.4%)	0.62
Hypertension	103 (17.3%)	19 (14.6%)	0.54

P-values calculated using independent samples T-test for continuous variables and Chi-square test for categorical variables.

**Table 2:** Table Comparative survival statistics for all failed implants (n=725).

Metric	No rescue group	Rescue therapy group
N	595	130
Mean Survival (Months)	26.9 (±39.7)	84.9 (±50.0)
Median Survival (Months)	8.7	88.8
Interquartile Range (IQR)	3.5-31.4	47.7-118.2
Min - Max (Months)	<1-260.2	4.2-200.1
Mann-Whitney U Test	Reference	p<0.001

2

**Table 3:** Table Comparative survival statistics for late failures (>12 months only).

Metric	No rescue group (>12mo)	Rescue therapy group (>12mo)
N	253	121
Median Survival (Months)	40.9	91.1
Survival Extension	Reference	+50.2 Months
Interquartile Range (IQR)	21.1-82.9	58.2-121.5
Mann-Whitney U Test	Reference	p<0.001

3

## Data Availability

The datasets used and/or analyzed during the current study are available from the corresponding author.
